# *Delftia acidovorans* Infections in Immunocompetent and Immunocompromised Hosts: A Case Report and Systematic Literature Review

**DOI:** 10.3390/antibiotics14040365

**Published:** 2025-04-01

**Authors:** Vincenzo Scaglione, Lucia Federica Stefanelli, Maria Mazzitelli, Leda Cattarin, Loreta De Giorgi, Elena Naso, Alberto Enrico Maraolo, Annamaria Cattelan, Federico Nalesso

**Affiliations:** 1 Infectious and Tropical Diseases Unit, Padua University Hospital, 35128 Padua, Italy; 2 Nephrology, Dialysis and Transplantation Unit, Padua University Hospital, 35128 Padua, Italymarialoreta.degiorgi@aopd.veneto.it (L.D.G.); elena.naso@aopd.veneto.it (E.N.); federico.nalesso@unipd.it (F.N.); 3 Section of Infectious Diseases, Department of Clinical Medicine and Surgery, University of Naples Federico II, 80131 Naples, Italy; albertomaraolo@mail.com; 4 Department of Molecular Medicine, University of Padova, 35128 Padova, Italy

**Keywords:** *Delftia acidovorans*, *Comamonas acidovorans*, infections, bacteremia, immunocompetent, immunocompromised, adult, children, review

## Abstract

*Delftia acidovorans* (*D. acidovorans*) is a non-fermentative, aerobic, Gram-negative bacillus typically found in environmental sources such as soil and water. Although considered an opportunistic pathogen, it has been implicated in both immunocompromised and immunocompetent individuals. This study presents a case of persistent cathether-related bacteraemia in a 61-year-old haemodialysis patient and offers a systematic literature review of similar cases. The patient, affected by end-stage kidney disease and dependent on a central venous catheter (CVC), presented with septic shock. Blood cultures confirmed *D. acidovorans*, resistant to aminoglycosides but sensitive to cephalosporins, piperacillin/tazobactam, and fluoroquinolones. Despite appropriate antibiotic therapy, bacteraemia persisted, prompting the use of taurolidine lock therapy when catheter removal was initially unfeasible. Blood cultures cleared after nine days, and the catheter was later replaced. A systematic review following PRISMA guidelines identified 21 additional cases of *D. acidovorans* bacteraemia. Most (76.2%) occurred in immunocompromised patients, particularly those with malignancies, chronic haemodialysis, or indwelling devices. Infections in immunocompetent individuals were typically associated with intravenous drug use or environmental exposure. Mortality was approximately 19%. Aminoglycoside resistance was consistent across most cases, while susceptibility to piperacillin/tazobactam, cephalosporins, and carbapenems was generally preserved. Given its resistance profile and ability to form biofilms, D. acidovorans poses a management challenge, particularly in catheter-associated infections. Rapid identification and targeted antimicrobial therapy are crucial. Adjunctive measures such as taurolidine lock therapy can be beneficial when device removal is not immediately possible.

## 1. Introduction

*Delftia acidovorans* (*D. acidovorans*) is a Gram-negative, aerobic, non-fermentative bacillus that shares several biological traits with species of the *Pseudomonas* genus [1]. Initially classified under the genus *Comamonas*, *D. acidovorans* was later reassigned based on phylogenetic and molecular analyses [2]. This bacterium is widely distributed in the environment and has been isolated from soil, polluted water, industrial effluents, and biofilms, demonstrating its resilience and adaptability to diverse ecological conditions [2,3]. Furthermore, *D. acidovorans* has been identified in raw milk and infected animals, expanding its known reservoir and emphasising its potential role in both environmental and zoonotic interactions [4]. It has also been detected in hospital settings, particularly in water systems and medical devices, raising concerns regarding its potential role as an opportunistic nosocomial pathogen [1,4]. Morphologically, *D. acidovorans* appears as slightly curved bacilli, occurring singly or in pairs. It demonstrates robust growth on various culture media, including blood agar, chocolate agar, and MacConkey agar, yet it does not ferment glucose, a characteristic that distinguishes it from other Gram-negative bacilli commonly encountered in clinical microbiology [5]. A key feature aiding in its phenotypical identification is its positive indole orange reaction, a biochemical trait useful in differentiation from related organisms [5,6].

Regarding molecular methods, 16S rRNA sequencing [7] and MALDI-TOF MS [8] importantly contribute to the identification of this pathogen.

Antimicrobial susceptibility testing (AST) for *D. acidovorans* is challenging because it is an uncommon pathogen, and clinical breakpoints are unavailable both for the European Committee on Antimicrobial Susceptibility Testing (EUCAST) and for the Clinical and Laboratory Standards Institute (CLSI) guidelines [9,10].

Considering the lack of species-specific guidelines for *D. acidovorans*, it is advisable to refer to the general recommendations for non-fermenting Gram-negative bacilli provided by CLSI and EUCAST, which support the use of broth microdilution as a reference method to perform AST due to its accuracy in determining minimum inhibitory concentrations (MICs) [9,10].

Notably, *D. acidovorans* exhibits a remarkable ability to form biofilm, a property that contributes to its persistence in both environmental and clinical settings and underscores its potential as an opportunistic pathogen [11]. Biofilm formation enhances its survival in harsh environments and increases its resistance to antimicrobial agents, further complicating its eradication from medical devices and hospital water systems [1,4,12]. Although infections caused by *D. acidovorans* are rare, they predominantly affect immunocompromised individuals, particularly those undergoing chemotherapy, living with underlying malignancies, or requiring prolonged use of medical devices such as central venous catheters (CVC) and prostheses [5,12]. The majority of reported infections are nosocomial in origin and include bloodstream infections, endocarditis, peritonitis, and pneumonia. However, sporadic cases in immunocompetent individuals have been documented, often associated with trauma or environmental exposure [5,13]. The opportunistic nature of *D. acidovorans* infections highlights the need for increased awareness and accurate diagnostic identification, particularly in hospitalised patients with underlying risk factors. Additionally, the bacterium has been implicated in polymicrobial infections, which can further complicate diagnosis and treatment [13].

A critical challenge in managing *D. acidovorans* infections is the distinctive antibiotic resistance profiles. As reported by Kang et al. [14], this bacterium exhibits intrinsic resistance to aminoglycosides, which are molecules frequently utilised in empirical therapy for Gram-negative infections, thus limiting treatment options [1].

The main mechanism of resistance to aminoglycosides is related to the oligopeptide transport system, which is important for the uptake of aminoglycoside antibiotics and decreases the amount of oligopeptide-binding protein (OppA), which limits the uptake of aminoglycosides and determines resistance to this class of molecules [14]. Other significant mechanisms of aminoglycoside resistance are related to aminoglycoside phosphotransferase (APH) and other aminoglycoside-modifying enzymes, such as aminoglycoside-acetylating enzyme (AAC) or aminoglycoside nucleotidyltransferase (ANT) [14].

Moreover, resistance to other commonly used antibiotics, including fluoroquinolones and some beta-lactams, has been observed in clinical isolates, further complicating therapeutic strategies [1]. Nevertheless, *D. acidovorans* generally remains susceptible to extended-spectrum cephalosporins, carbapenems, and piperacillin–tazobactam, underscoring the importance of species-level identification for effective antimicrobial therapy [1].

The primary aim of this study was to synthesise and analyse existing evidence regarding the clinical outcomes of *D. acidovorans* bloodstream infections in both immunocompromised and immunocompetent hosts. In addition, we present a case report from our clinical practice to provide further insights into the microbiological and clinical characteristics of this rare pathogen. By integrating a comprehensive review of the literature with real-world clinical experience, we seek to enhance the understanding and awareness of this underrecognized microorganism, thereby facilitating more effective diagnosis and management strategies in clinical settings.

## 2. Case Description

This is the case with a 61-year-old homeless gentleman. His medical history included an allergy to paracetamol and an adverse drug reaction to quinine. Regarding comorbidities, the patient was affected by hypertension, which caused a stroke in 2012, resulting in secondary epilepsy. Moreover, during childhood, the patient developed malaria and yellow fever. In 2017, he received isoniazid for latent tuberculosis for nine months. His medical treatment included valproate, acetylsalicylic acid, sevelamer carbonate, nebivolol, epoetin, amlodipine, doxazosin, and pantoprazole.

This patient developed end-stage kidney disease (ESKD) due to nephroangiosclerosis in 2014; therefore, he was receiving haemodialysis via a tunnelled CVC since multiple attempts at vascular access formation with a failed left brachiocephalic native arteriovenous fistula (AV) failed in the past (2015, 2016, and 2017). For this reason, the CVC was the most suitable vascular access. However, due to his poor life condition, he experienced recurrent catheter-related infections, which were managed by using a prolonged antibiotic course and device removal (the latter bacteraemia caused by Escherichia coli occurred in 2023).

On 27 March 2024, while receiving routine outpatient dialysis, the patient developed a fever of 39.8 °C and chills, with severe hypotension, a heart rate of 130 beats per minute, and blood pressure of 58/ 58/40 mmHg. Therefore, he was promptly admitted because of suspicion of septic shock, and after performing blood culture, treatment with meropenem and vancomycin was started. Laboratory tests at the time of admission revealed an elevated C-reactive protein (CRP) of 176.60 mg/L and procalcitonin (PCT) level of 369 µg/L; electrocardiogram and chest X-ray were unremarkable. Blood cultures obtained from both the CVC and peripheral vein (27 March 2024) were positive for a Gram-negative rod identified as *D. acidovorans*, which was resistant to aminoglycosides but sensitive to cephalosporins, fluoroquinolones, and piperacillin–tazobactam. Therefore, vancomycin was discontinued, and meropenem was switched to piperacillin–tazobactam. Despite clinical and laboratory improvement, follow-up blood cultures persisted to be positive 3, 5, 7, 9 and 11 days after the start of the antibiotic treatment. Transthoracic echocardiography was performed, and infectious endocarditis related to CVC was excluded. Positron emission tomography-computed tomography excluded other sources of infection.

Even if the patient met the criteria for catheter removal, this was not immediately possible due to concomitant partial thrombosis of the vascular access and the complexity vascular access history of the patient, characterised by several native AV failures. For this reason, CVC was left in place, and lock therapy with Taurolock Hep 500 was started as rescue therapy for the catheter (4 April 2024). The patient was afebrile during the 2 weeks of lock therapy, and blood cultures became negative 9 days after their implementation, alongside the antimicrobial treatment (13 April 2024). To minimise the risk of reinfection, CVC was removed and replaced on 23 April 2024. A follow-up transthoracic echocardiogram performed four weeks later was unremarkable. The patient was discharged after replacement of the CVC and 4 weeks of antimicrobial treatment.

## 3. Materials and Methods

The literature search was performed according to Preferred Reporting Items for Systematic Reviews and Meta-Analyses (PRISMA) guidelines [15]. The following databases were systematically queried: PubMed/MEDLINE/EMBASE. No age or geographical restrictions were applied, and only papers published in English were included. Studies published backward on 31 December 2024 were considered. The search strategy included the following Medical Subject Headings (MeSH), Emtree terms, keywords, and Boolean operators: (“*Delftia acidovorans*” [MeSH Terms] OR “*Delftia acidovorans*” [All Fields] OR “*Comamonas acidovorans*” [All Fields] OR “*Pseudomonas acidovorans*” [All Fields]) AND (“infection” [MeSH Terms] OR “infection” [All Fields] OR “bacteremia” [MeSH Terms] OR “bacteremia” [All Fields] OR “sepsis” [MeSH Terms] OR “sepsis” [All Fields] OR “septicemia” [All Fields] OR “abscess” [MeSH Terms] OR “abscess” [All Fields] OR “endocarditis” [MeSH Terms] OR “endocarditis” [All Fields] OR “meningitis” [MeSH Terms] OR “meningitis” [All Fields] OR “pneumonia” [MeSH Terms] OR “pneumonia”[All Fields] OR “urinary tract infections” [MeSH Terms] OR “urinary tract infection” [All Fields] OR “catheter-related infections” [MeSH Terms] OR “catheter-related infection” [All Fields]). Original case reports, case series, observational studies, clinical trials, and review articles that clearly reported infections caused by *Delftia acidovorans* were included, even considering immunocompetent and/or immunocompromised hosts, with their clinical presentation, microbiological identification, management strategies, and patient outcomes. Articles without clear identification of *Delftia acidovorans*, studies reporting environmental or microbiological-only assessments without clinical patient data, conference abstracts without full-text availability, and animal studies were excluded. Two independent reviewers (MM and AEM) screened titles and abstracts for potential relevance. Full-text articles were then assessed independently against inclusion/exclusion criteria. Discrepancies were resolved by consensus or consultation with a third reviewer (VS). Data were extracted into a standardised form, capturing authorship, year of publication, country, host immune status, clinical manifestations, microbiological identification methods, antimicrobial susceptibility profiles, treatment regimens, and patient outcomes. In the case of missing information or data reported in the papers in aggregate form, we contacted the corresponding authors of each work, requesting the information. In case of no response, the article was mentioned in the review but removed from the data analysis because it was not possible to find the information of the individual patients. References from included studies were cross-checked manually to identify any additional relevant literature. Duplicates were eliminated using EndNote 20 reference management software (Clarivate; Philadelphia, PA, USA). The quality of included studies was assessed using tools appropriate for case reports and observational studies (e.g., JBI Critical Appraisal Checklist for Case Reports or Newcastle–Ottawa Scale). The systematic search and selection process is summarised following PRISMA guidelines, presented in the form of a PRISMA flow diagram within the manuscript. The study protocol of this review was not registered.

## 4. Results

**Figure 1:**
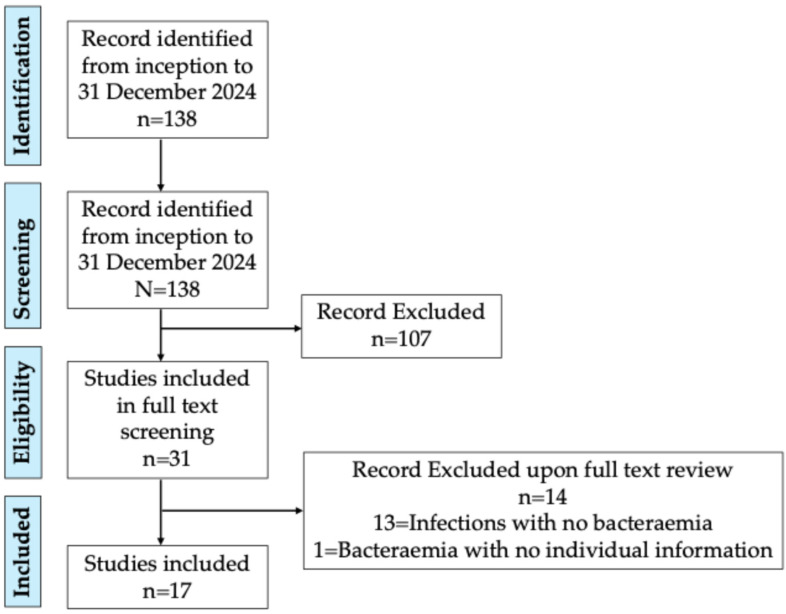
PRISMA Diagram flow. The initial search identified 138 studies, of which 107 were excluded because they did not address infections. Thirty-one studies were reviewed in full, and seventeen were ultimately included in the quantitative analysis, as they met the eligibility criteria, describing cases of bacteraemia due to *Delftia acidovorans*. Thirteen studies describing *D. acidovorans* infections without concurrent bacteraemia (e.g., pneumonia, eye infections) were excluded. One study was excluded because its aggregated data on 17 bacteraemia cases could not be analysed individually.

Figure 1 reports the diagram flow showing the results of the literature search.

The first search identified 138 studies; 107 were removed because they did not treat cases of infections, 31 were included for a full review, and 17 were included for the quantitative analysis, since they met all eligible criteria by describing cases of bacteraemia due to *D. acidovorans*. Thirteen studies were excluded because they described cases of *D. acidovorans* infections for which there was no concurrent bacteraemia (pneumonia, eye infections, etc.). Another study [1] was excluded because even if mentioning 17 cases of bacteraemia, the data were reported as aggregated, and it was not possible to retrieve individual information for analysis. The 17 included studies that described 21 cases and our patient cases are summarised in Table 1.

They were all retrospective descriptions of case reports and series. The first case was reported in 1990 by Horovitz et al., who described a case of infectious endocarditis caused by *D. acidovorans* in an adult lady injected with drugs [23]. Most cases (14/21, 66.7%) were adult, while seven were children whose age ranged from the neonatal period to 11 years. In adults, all age groups were represented, ranging from 27 to 93 years. Male gender was the most represented in adults (10/14, 71.4%), while the genders in children were almost equally distributed (three females and four males).

Overall, 16/21 (76.2%) patients were immunocompromised, 10/14 (71.4%) in adults and 6/7 (85.7%) in children. The most common risk factor for immunosuppression in children was malignancy (3/7, 42.8%). Overall, 6/21 (28.6%) bacteraemia cases were related to intravascular indwelling devices. As in our patient (#22), chronic haemodialysis was a risk factor for bacteraemia in one child (#5) and in 3/14 (21.4%) adults. Active intravenous drug use was the likely cause of bacteraemia in 3/14 (21.4%) adults. Septic shock at admission was present in 2/21 (9.5%) described cases and in our patient. The AST levels of the isolates are summarised in Table 2. Where the data were available, the isolated strains were, in most cases, susceptible to piperacillin–tazobactam, third and fourth generation cephalosporins, fluoroquinolones, trimetoprim/sulfametoxazole, and carbapenems. Conversely, as expected, resistance to aminoglycosides was present in all cases except for an unusual case reported by Hagiya et al. [29], where the isolated strain was labelled as “sensitive” to amikacin by these authors with a MIC value of 16 calculated using a semi-automated method.

Regarding antimicrobial treatment, no information was available for two cases (#16 and #17). Empirical antibiotic therapy was appropriate according to the AST in 4/19 (21.0%) cases. Death occurred in 4/21 (19%) cases, 1 in children (14.2%) for whom the empirical antibiotic therapy was not active against *D. acidovorans* and 3 in adults (21.4%).

## 5. Discussion

To the best of our knowledge, this is the first comprehensive assessment of the available evidence of *D. acidovorans* bacteraemia, a rare occurrence whose identification has been made easier by modern tools such as MALDI-TOF mass spectrometry [8].

Our literature review indicates that while the majority of reported cases of *D. acidovorans* bacteraemia involve immunocompromised hosts, there is increasing evidence documenting infections in immunocompetent individuals. This dichotomy underscores the pathogen’s adaptability and capacity to exploit various host vulnerabilities, including intravenous drug use, systemic immunosuppression, or localised conditions such as biofilm formation on medical devices. As noted, immunocompromised hosts, particularly those undergoing chemotherapy, chronic haemodialysis, or implanted vascular devices, constitute a significant proportion of cases. This observation aligns with the findings of Ender et al. [17] and Castagnola et al. [16], who documented catheter-associated bacteraemia in both paediatric and adult populations. The propensity for biofilm formation on indwelling devices, as described by Nejadnik et al. [33], further emphasises the clinical significance of pathogens.

Our review and case identified 22 patients with bacteraemia caused by *D. acidovorans*. Hojgarrd et al. identified 17 patients with bacteraemia caused by *D. acidovorans* in their experience [1]. However, we were unable to retrieve all the required information from this review.

The inherent resistance of *D. acidovorans* to aminoglycosides necessitates species-specific AST to guide therapy. Moreover, since the lack of specific guidelines by CLSI or EUCAST institutions [9,10], the use of an accurate method, such as broth microdilution, should be pursued to achieve reliable information. For example, Hagiya et al. [29] reported sensitivity for amikacin in their isolated strains; however, these authors reported a MIC value of 16, which is on the edge of the breakpoint, and also used a semi-automated method to calculate the MIC, which may be inaccurate for non-fermentative Gram-negative bacilli such as *D. acidovorans*, thus increasing the risk of inappropriate therapy.

Despite the limited number of cases, information regarding treatment is not readily available, and a literature review confirms that piperacillin–tazobactam, cephalosporins, and fluoroquinolones remain effective first-line agents for most strains; in some instances, carbapenems have been effectively utilised.

Despite the lack of molecular phylogenetic analysis of our isolated strain, our case reinforces the importance of the prompt initiation of targeted therapy. Transitioning to piperacillin–tazobactam led to clinical and microbiological resolution in the absence of endocarditis or alternative foci of infection.

Our review showed a high variability in therapeutic regimens; therefore, it remains unclear whether monotherapy with an active antibiotic is an appropriate approach or if combination therapy may be warranted in severe or complicated cases. Despite the rationale for using an empirical combination therapy being supported by the severity of clinical cases in the first place, some combinations may not be active, and more research is needed in this field to prove that combination therapy (either empirical or targeted) may be more effective than monotherapy.

However, persistent bacteraemia despite appropriate therapy, as observed in our patient, suggests a critical role for adjunctive measures, such as taurolidine lock therapy.

Indeed, in our case, when catheter removal was not immediately feasible, taurolidine was crucial for resolving the bacteraemia. Taurolidine is a broad-spectrum antimicrobial agent with unique antibiofilm and anti-inflammatory properties, rendering it a promising option for managing catheter-related infections, particularly in scenarios where device removal is not feasible. The mechanism of action involves the release of reactive methylol groups that interact with bacterial cell walls and toxins, thereby disrupting biofilm formation and reducing microbial virulence [33]. This feature is particularly advantageous in patients with long-term indwelling catheters, such as CVCs, where biofilm-mediated infections present a significant clinical challenge. Several studies have demonstrated the efficacy of taurolidine in preventing catheter-related bloodstream infections, especially in vulnerable populations such as paediatric oncology patients [34] and in patients undergoing haemodialysis, where it has been shown to decrease the incidence of infections and improve catheter longevity [35].

Taurolidine has emerged as an efficacious supplementary therapy in scenarios in which catheter extraction is precluded by medical or logistical impediments. This agent not only diminishes the infection risk but also contributes to the preservation of catheter functionality. A cohort investigation conducted by Brescia et al. showed that taurolidine lock solutions may be a suitable solution, obviating the need for catheter removal [36]. Notwithstanding its limited accessibility in certain geographical areas, taurolidine is a viable alternative for addressing catheter infections in high-risk cohorts [34]. Thaurolodine’s clinical utility may be limited by potential adverse effects, including local irritation, allergic reactions, and occasional catheter occlusion, along with concerns regarding its higher costs compared to alternative solutions. Consequently, alternative strategies such as citrate-based locks or antibiotic lock solutions (e.g., gentamicin–heparin or vancomycin–heparin locks) have been explored, often demonstrating comparable effectiveness, improved tolerability, and greater cost-efficiency in clinical settings.

The mortality rates associated with *D. acidovorans* bacteraemia vary. This analysis revealed a case fatality rate of approximately 19%, which is consistent with earlier observations by Højgaard et al. [1]. Mortality-contributing factors include delayed diagnosis, suboptimal initial antibiotic regimens, and underlying comorbidities. The administration of appropriate antimicrobial therapy demonstrates a robust correlation with favourable outcomes, even in instances of septic shock, as corroborated by this review and previous investigations [26,30].

Healthcare practitioners should exercise heightened vigilance for *D. acidovorans* in patients presenting with persistent bacteraemia, particularly those with implanted medical devices or extensive healthcare exposure. The implementation of rapid identification methodologies utilising molecular techniques, in conjunction with susceptibility-guided therapeutic approaches is crucial for achieving optimal clinical outcomes. Moreover, the consideration of adjunctive strategies, such as taurolidine lock therapy, is warranted in complex cases where device extraction presents substantial challenges.

Although uncommon, *D. acidovorans* infections constitute a significant clinical entity necessitating increased awareness among medical professionals. The organism’s capacity to induce persistent and recurrent infections underscores the importance of comprehensive management strategies, encompassing targeted antimicrobial interventions and innovative adjunctive measures such as taurolidine locks. Our study is affected by several limitations, considering the retrospective nature of the data, the lack of molecular phylogenetic analysis of the isolated strain, and the heterogeneity of case reports analysed in the context of a very uncommon pathogen.

## 6. Conclusions

Although rare, *D. acidovorans* represents an emerging opportunistic pathogen capable of causing persistent and potentially life-threatening infections, particularly in immunocompromised individuals and those with indwelling medical devices. Our case and systematic review underscore the importance of accurate and timely identification, species-specific antimicrobial susceptibility testing, and individualised therapeutic strategies, especially given the organism’s intrinsic resistance to aminoglycosides and its biofilm-forming capacity. While most isolates remain susceptible to piperacillin–tazobactam, cephalosporins, and carbapenems, clinical outcomes are closely tied to the appropriateness of empirical and targeted therapy. Adjunctive treatments, such as taurolidine lock therapy, offer promising alternatives when catheter removal is not immediately feasible. Increased awareness and reporting of *D. acidovorans* infections are essential to inform best practices, and further research is warranted to define standardised treatment approaches and explore the pathogen’s resistance mechanisms more comprehensively.

## Figures and Tables

**Table 1:** Summary of cases of *D. acidovorans* bacteraemia. Our research identified a high frequency of malignancies in both paediatric and adult patients. However, the case of our patient is somewhat uncommon, as the primary risk factor is the prolonged use of CVC to receive dialysis treatment due to ESKD.

Authors and Year of Publication	Patient ID	N Cases	Sex	Age	Immunocompromised (Yes/No)	Comorbidities and Risk Factors	Type of Infection	Septic Shock (Yes/No)	Treatment	Appropriate by AST? Y/No	Outcome
**Paediatric population**											
Castagnola et al., 1994 [16]	#1	1	Male	9	Yes	Non-Hodgkin lymphoma	Catheter-related bloodstream infection	No	Ceftazidime and vancomycin, then amikacin for 12 days	Y	Favourable
Ender et al., 1996 [17]	#2	1	Female	4	Yes	Metastatic neuroblastoma and persistent neutropenia, autologous stem transplant, recurrent urinary tract infections	Catheter-related bloodstream infection	No	Ceftazidime + ciprofloxacin 14 days	Y	Favourable
Oliver et al., 2005 [18]	#3	1	Male	10	No	None	Bacteraemia	No	Piperacillin/tazobactam and metronidazole, then imipenem + ciprofloxacin for 8 days, then cefepime for 3 days	Y	Favourable
Kawamura et al., 2010 [19]	#4	1	Female	11	Yes	Metastatic neuroblastoma and persistent neutropenia, autologous stem transplant	Catheter-related bloodstream infection	No	Cefpirome for 10 days, then panipenem/betamipron for 7 days	Y	Favourable
Chotikanatis K et al., 2011 [20]	#5	1	Female	10	Yes	Haemodialysis	Recurrent catheter-related bloodstream infection	No	Cefepime 14 days Ceftazidime due to the onset of resistance for 21 days	Y	Favourable
Agarwal et al., 2023 [21]	#6	1	Male	Newborn	Yes	None	Bacteraemia	Yes	Ampicillin + Gentamycin	N	Death
Alam et al., 2023 [22]	#7	1	Male	Newborn	Yes	Anal stenosis	Bacteraemia+pneumonia	Yes	Meropenem + ciprofloxacin	Y	Favourable
**Adult population**											
Horowitz H et al., 1990 [23]	#8	1	Female	42	No	Intravenous drug use	Infective endocarditis	No	Ceftazidime + penicillin, then ciprofloxacin + amikacin	Y	Death
Lair et al., 1996 [24]	#9	1	Male	27	Yes	AIDS	Bacteraemia		Imipenem for 1 week + amikacin for 17 days	Y	Favourable
Perla et al., 2005 [25]	#10	1	Male	35	No	Intravenous drug use	Bacteraemia	No	Cefotaxime + gentamycin, then imipenem, then levofloxacine for 10 days	Y	Favourable
Kam et al., 2012 [26]	#11	1	Male	93	Yes	Prostate hyperplasia, obstructive uropathy, chronic bronchitis, hypertensive cardiovascular disease	Bacteraemia associated with an ascendent urinary tract infection	No	Flomoxef, then amikacin, then cefoperazone/sulbactam, then imipenem for 14 days	Y	Favourable
Lang et al., 2012 [27]	#12	1	Male	65	Yes	NK cell lymphoma	Catheter-related bloodstream infection	No	Piperacillin/tazobactam+gentamycin, then imipenem/cilastatin+teicoplanin	Y	Favourable
Mahmood S et al., 2012 [28]	#13	1	Male	30	No	Intravenous drug use, hepatitis C, and post-traumatic stress disorder	Infective endocarditis	No	Vancomyicin + piperacillin/tazobactam then ceftriaxone + surgery	Y	Favourable
Hagiya et al., 2013 [29]	#14	1	Female	46	No	Organophosphorus poisoning	Bacteraemia	No	ampicillin/sulbactam 9 days, then meropenem 3 days and piperacillin/tazobactam 4 days	N	Favourable
Singh et al., 2022 [30]	#15	1	Female	29	Yes	Breast cancer	Catheter-related bloodstream infection	No	Meropenem + teicoplanin	Y	Death
Backman et al., 2023 [31]	#16	2	Male	35	Yes	Membranoproliferative glomerulonephritis	Bacteraemia	No	Not available	Y	Favourable
	#17		Male	50	Yes	Diabetes	Bacteraemia	No	Not available		Favourable
Tsung-Lung et al., 2024 [32]	#18	4	Female	89	Yes	2 comorbidities	Bacteraemia	No	Ampicillin/sulbactam	N	Favourable
	#19		Male	51	Yes	3 comorbidities	Bacteraemia	No	Amikacin	N	Favourable
	#20		Male	78	Yes	1 comorbidity	Bacteraemia	No	Imipenem	Y	Favourable
	#21		Male	89	Yes	Rectal cancer	Bacteraemia	No	Ciprofloxacin	Y	Death
Our case, 2024	#22	1	Male	61	Yes	Chronic haemodialysis	Catheter-related bloodstream infection	Yes	Meropenem, then piperacillin/tazobactam	Y	Favourable

**Table 2:** Resistance profile of all *D. acidovorans* isolates.

		Antibiotics																					
Authors and Year of Publication	Patient ID	Penicillin	Ampicillin/Sulbactam	Amoxicillin/Clavulanate	Piperacillin/Tazobactam	Cefuroxime	Cefepime	Ceftriaxone	Cefpodoxime	Ceftazidime	Trimetoprim/Sulfametoxazole	Gentamycin	Tobramycin	Tetracycline	Ciprofloxacin	Moxifloxacin	Nitrofurantoin	Imipenem	Meropenem	Cloramphenicol	Aztreonam	Colisitin	Amikacin
Castagnola et al., 1994 [16]	#1	R	I	-	S	S	S	S	S	S	S	R	-	-	S	-	-	S	S	-	-	-	-
Ender et al., 1996 [17]	#2	-	I	-	S	S	S	S	-	S	S	R	-	-	S	-	-	S	S	-	S	-	R
Oliver et al., 2005 [18]	#3	-	-	-	S	S	S	S	S	S	S	R	R	-	S	-	-	S	S	-	S	-	-
Kawamura et al., 2010 [19]	#4	-	-	-	S	-	S	-	S	S	S	R	R	-	S	-	-	S	S	-	S	-	-
Chotikanatis K et al., 2011 [20]	#5	-	I	-	S	S	S	-	-	S	S	R	-	-	S	-	-	S	S	-	S	-	R
Agarwal et al., 2023 [21]	#6	-	-	-	S	S	S	S	S	S	-	R	-	-	R	-	-	S	S	-	-	R	R
Alam et al., 2023 [22]	#7	-	-	-	R	-	R	-	-	S	-	R	-	-	S	-	-	-	S	-	-	R	R
Horowitz H et al., 1990 [23]	#8	-	-	-	-	R	S	S	-	S	S	R	R	-	S	-	-	-	-	-	S	-	R
Lair et al., 1996 [24]	#9	-	-	-	S	-	-	-	-	S	-	R	-	-	-	-	-	S	-	-	-	R	R
Perla et al., 2005 [25]	#10	-	-	-	S	S	-	S	-	S	S	R	I	-	S	-	-	S	-	-	-	-	-
Kam et al., 2012 [26]	#11	R	R	R	R	-	-	-	-	-	-	R	R	-	R	R	-	S	S	-	-	-	R
Lang et al., 2012 [27]	#12	-	-	-	-	-	-	-	-	-	-	R	R	-	-	-	-	S	S	-	-	-	R
Mahmood S et al., 2012 [28]	#13	-	-	-	S	-	R	S	-	S	S	R	R	S	S	-	-	S	S	-	-	-	R
Hagiya et al., 2013 [29]	#14	-	-	-	S	-	R	-	-	S	S	R	-	-	S	-	-	S	S	-	S	-	S
Singh et al., 2022 [30]	#15	-	-	-	S	-	-	-	-	S	-	-	-	-	S	-	-	-	S	-	S	R	R
Backman et al., 2023 [31]	#16	-	-	-	-	-	-	-	-	-	-	-	-	-	-	-	-	-	-	-	-	-	-
Backman et al., 2023 [31]	#17	-	-	-	-	-	-	-	-	-	-	-	-	-	-	-	-	-	-	-	-	-	-
Tsung-Lung et al., 2024 [32]	#18	-	-	-	S	-	-	-	-	S	-	R	-	-	-	-	-	S	S	-	-	-	R
Tsung-Lung et al., 2024 [32]	#19	-	-	-	S	-	-	-	-	S	-	R	-	-	-	-	-	S	S	-	-	-	R
Tsung-Lung et al., 2024 [32]	#20	-	-	-	S	-	-	-	-	S	-	R	-	-	-	-	-	S	S	-	-	-	R
Tsung-Lung et al., 2024 [32]	#21	-	-	-	S	-	-	-	-	S	-	R	-	-	-	-	-	S	S	-	-	-	R
Our case	#22	-	-	-	S	S	S	S	S	S	S	R	-	-	S	-	-	S	S	S	-	-	R

S = Susceptible, I = Intermediate, R = Resistant. “-” = not available or not tested. The term Intermediate may represent a non-susceptible phenotype. Not all isolates were tested against all antibiotics.

## Data Availability

The datasets generated during the current review are available from the corresponding author upon reasonable request. All data analysed are included in the corresponding published articles, as reported in Table 1.

## Abbreviations

The following abbreviations are used in this manuscript:

AAC	aminoglycoside-acetylating enzyme
ANT	aminoglycoside nucleotidyltransferase
APH	aminoglycoside phosphotransferase
AST	antimicrobial susceptibility testing
CVC	Central Venous Catheter
AV	arteriovenous fistula
CLSI	Clinical and Laboratory Standards Institute
CRP	C-reactive protein
ESKD	end-stage kidney disease
EUCAST	European Committee on Antimicrobial Susceptibility Testing
MIC	minimum inhibitory concentration
OppA	Oligopeptide-binding protein
PCT	procalcitonin
